# Effect of calcium-channel blockers on the risk of active tuberculosis and mortality: systematic review and meta-analysis

**DOI:** 10.3389/fphar.2024.1298919

**Published:** 2024-01-18

**Authors:** Edinson Dante Meregildo-Rodriguez, Martha Genara Asmat-Rubio, Victor Hugo Bardales-Zuta, Gustavo Adolfo Vásquez-Tirado

**Affiliations:** ^1^ Escuela de Medicina, Universidad César Vallejo, Trujillo, Peru; ^2^ Escuela de Posgrado, Universidad Privada Antenor Orrego, Trujillo, Peru; ^3^ Escuela de Medicina, Universidad Privada Antenor Orrego, Trujillo, Peru

**Keywords:** calcium channel blockers, tuberculosis, mortality, systematic review, meta-analysis

## Abstract

**Introduction:** Recent studies suggest that calcium channel blockers (CCBs) could reduce the risk of active tuberculosis and improve clinical outcomes. We aimed to synthesize the evidence regarding the effect of CCBs on the risk of developing active tuberculosis and mortality.

**Methods:** We systematically searched for observational studies and clinical trials published in six databases until 31 August 2023, following a PECO/PICO strategy.

**Results:** We included eight observational studies, 4,020,830 patients, among whom 241,761 had diabetes mellitus and 30,397 had active tuberculosis. According to our results, CCBs reduce the risk of developing active tuberculosis by 29% (RR 0.71; 95% CI 0.67–0.75) in patients with and without diabetes mellitus. However, CCBs do not show any benefit in terms of tuberculosis-related mortality (RR 1.00; 95% CI 0.98–1.02). For both outcomes, no statistical heterogeneity was found (I^2^ = 0, *p* > 0.10). This protective effect of CCBs on the risk of active tuberculosis remained independent of the type of patient (with diabetes mellitus vs. general population) or the class of CCB administered (DHP-CCB vs. non-DHP-CCB) (test for subgroup differences I^2^ = 0, *p* > 0.10). However, this beneficial effect was more significant among the general population (RR 0.70; 95% CI 0.66–0.74) compared to patients with diabetes mellitus (RR 0.72; 95% CI 0.61–0.86) and among those patients treated with DHP-CCBs (RR 0.69; 95% CI 0.63–0.74) compared to patients treated with non-DHP-CCBs (RR 0.72; 95% CI 0.67–0.78).

**Conclusion:** CCBs may reduce the risk of active TB in patients with diabetes and the general population. On the contrary, CCBs do not seem to have a protective effect on tuberculosis-related mortality. However, more evidence is still needed. We recommend developing clinical trials to verify these findings, including more diverse populations.

**Systematic Review Registration:** [https://www.crd.york.ac.uk/prospero/display_record.php?RecordID=352129]

## 1 Introduction

Tuberculosis (TB) is a formidable global public health problem that claims more than one million lives yearly. Indeed, TB stands as one of the leading causes of morbidity and mortality stemming from infectious diseases worldwide (World Health Organization, 2022b). The World Health Organization’s Global Plan aims to eradicate TB by 2050 (Global tuberculosis report. World Health Organization, 2022). However, achieving this is challenging due to the inadequate rate of decline in tuberculosis cases. An effective TB vaccine remains elusive, and TB treatment necessitates the prolonged use of multi-drug regimens, heightening the risk of adverse effects, compliance (adherence) issues, and drug resistance (World Health Organization, 2022a; Global tuberculosis report. World Health Organization, 2022). Therefore, a preventive strategy against tuberculosis is preferred (World Health Organization, 2022a).

Drugs rapidly kill most *Mycobacterium tuberculosis* (MTB) bacilli. However, eliminating persistent and drug-tolerant subpopulations requires prolonged treatment (Sebastian et al., 2022). The differential sensitivity of MTB to drugs is determined, at least in part, by the interaction between the bacilli and the various macrophage populations of the host (Mitini-Nkhoma et al., 2021). Therefore, to design better treatment regimens for TB, we must understand the heterogeneity and modulate the divergent responses of MTB bacilli within macrophages (Mitini-Nkhoma et al., 2021). Consequently, identifying new treatment strategies against this disease should be a public health priority. However, developing drugs *de novo* is a long and expensive process. An alternative approach to expedite new TB therapies is to repurpose existing drugs developed for other therapeutic purposes if they show to possess anti-TB activity (Gupta et al., 2013; Demitto et al., 2015; Mitini-Nkhoma et al., 2021).

There is growing interest in utilizing immunomodulators to complement the existing anti-TB drugs by enhancing the host’s anti-mycobacterial responses. Statins, beta-blockers, and calcium channel blockers (CCBs) have been reported as some of the most promising host-directed therapies (Lee et al., 2015a; Tahir et al., 2020; Dutta et al., 2021; Mitini-Nkhoma et al., 2021; Meregildo-Rodriguez et al., 2022a; Cubillos-Angulo et al., 2022). Additionally, certain ion channel blockers interfere with the activity of mycobacterial efflux pumps (Kristiansen and Amaral, 1997; Gupta et al., 2013; Mitini-Nkhoma et al., 2021). Iron acquisition is essential for several intracellular pathogens, including MTB. Iron availability favors mycobacterial growth and promotes infection, replication, and progression to clinical disease and death (Biswas et al., 2008; Gupta et al., 2009; Kondratskyi et al., 2015). Patients with tuberculosis often develop anemia of chronic disease. This inflammatory response results in iron dysregulation, where the body sequesters iron to prevent it from being used by organisms such as MTB. Therefore, TB patients may have an elevated ferritin level and a corresponding low serum iron and transferrin level (Lee et al., 2021; Isanaka et al., 2012).

On the other hand, iron deprivation can reduce the viability and replication of MTB, potentially preventing the reactivation of latent TB. *In vitro* studies have shown that L-type voltage-gated calcium channels provide an alternative pathway for iron entry into different cell types (Lee et al., 2021; Oudit et al., 2003; Gaasch et al., 2007). CCBs target the L-type voltage-gated calcium channel, and several studies have found that CCBs can decrease plasma iron levels (Oudit et al., 2003; Ludwiczek et al., 2007; Mainous et al., 2012; Fernandes et al., 2013). CCBs reduce iron availability, an essential mineral for intracellular pathogens, including MTB. However, it is still unclear whether CCB administration modifies the risk of active tuberculosis in the clinical setting (Lee et al., 2021). The available evidence is contradictory, with some studies suggesting that CCBs could reduce the risk of developing active TB (Lee et al., 2021; Lee et al., 2015a; Lee et al., 2015b), while other studies have not confirmed these findings (Chidambaram et al., 2021). Therefore, this study aims to clarify whether using CCBs reduces the risk of developing active tuberculosis.

## 2 Materials and methods

This systematic review adhered to the recommendations outlined in the Cochrane Handbook for Systematic Reviews (Higgins et al., 2023), PRISMA (Page et al., 2021), and AMSTAR 2 (Shea et al., 2017) guidelines. The protocol was registered in PROSPERO (CRD42022352129).

### 2.1 Search strategy

We comprehensively searched various databases, including MEDLINE (PubMed), Scopus, EMBASE, Web of Science, ScienceDirect, and Google Scholar. We screened each database using controlled language terms (MeSH, Emtree, etc.), free terms, and their synonyms, combined with Boolean operators, following the PECO/PICO strategy. Keywords primarily focused on exposure, such as “calcium channel blockers,” OR “calcium antagonist,” OR “dihydropyridine,” OR “non-dihydropyridine,” and outcome-related terms like “tuberculosis,” OR “active tuberculosis,” OR “active TB disease,” OR “mortality.” Additionally, we performed manual secondary searches of references in relevant studies and review articles. There were no restrictions on language or publication year. The search strategy is provided in the Supplementary Table S1.

### 2.2 Inclusion and exclusion criteria

Our search included observational studies and randomized controlled trials (RCTs) published from inception until 31 August 2023. We excluded case reports, case series, and duplicated publications. All articles resulting from the primary and secondary searches were initially organized using Zotero^®^ 6.0.15. After removing duplicates, these documents were imported into the Rayyan^®^ tool, screened, and individually examined by three blinded and independent researchers (MA-R, VB-Z, and GV-T). The initial selection of studies was made by consensus, and in case of disagreement, a fourth researcher served as the arbitrator (EM-R). All collected articles were evaluated using the terms of the PECO/PICO strategy and the inclusion and exclusion criteria.

### 2.3 Study selection and data extraction

**FIGURE 1:**
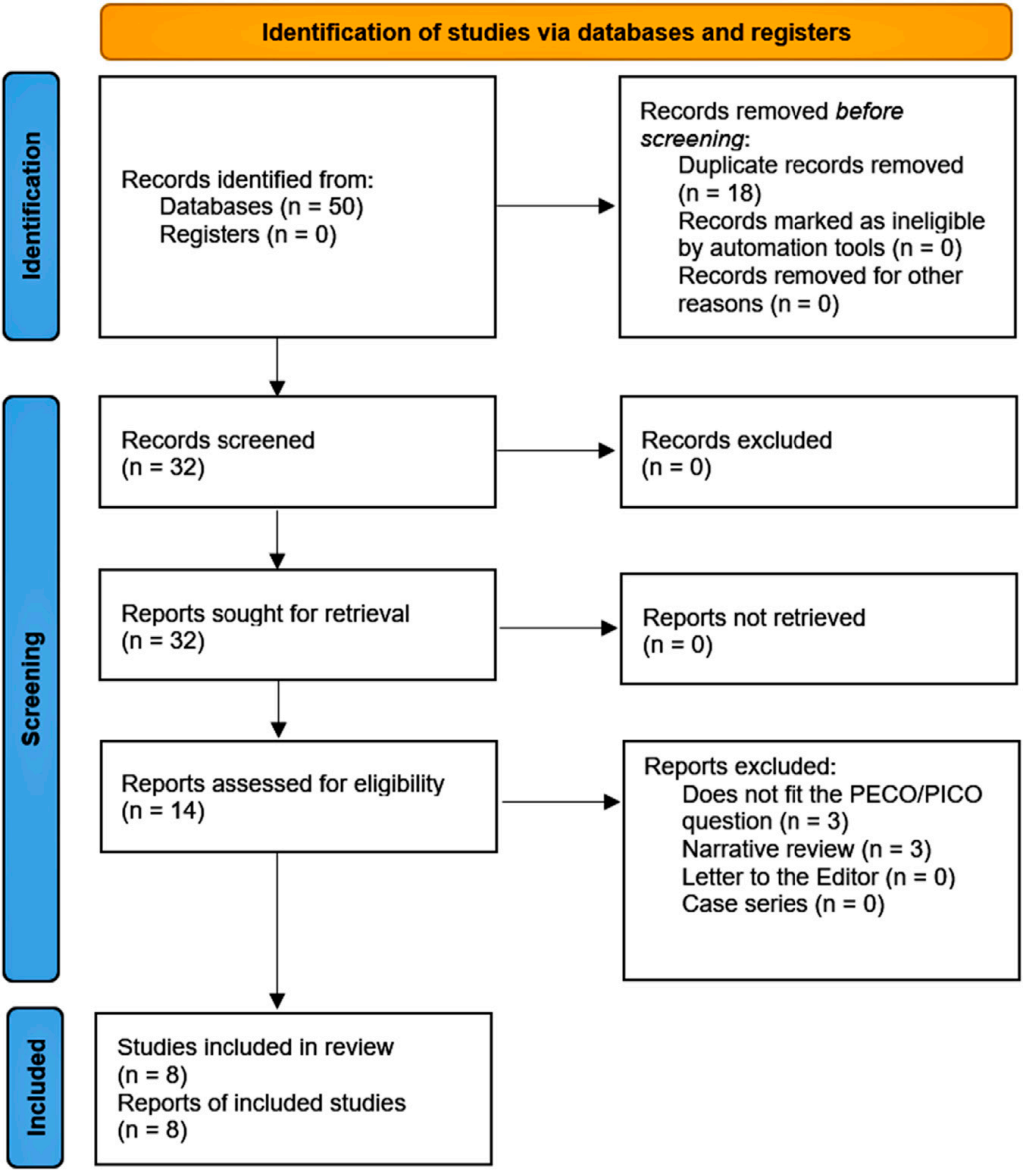
PRISMA flowchart.

The selected articles were exported to a spreadsheet for a second full-text screening. The study selection process is detailed in Figure 1. For data extraction, the same three blinded and independent researchers who performed the selection process examined articles and collected the relevant details of the study, including the authors, country and year of publication, clinical and epidemiological characteristics of the population, number of patients and cases (events), measures of association, confounding factors, and the most relevant outcomes. For dichotomous and time-to-event variables, we compiled odds ratios (OR), relative risks (RR), and hazard ratios (HR) with 95% confidence intervals (95% CI). If critical data were missing, at least two emails were sent to the corresponding authors. Data from each paper were extracted and recorded in a spreadsheet. In case of a discrepancy, a fourth researcher (EM-R) was invited to solve it if necessary.

### 2.4 Data synthesis, meta-analysis, and meta-regression

We conducted this meta-analysis using R^®^ 4.2.226 and RStudio^®^ 2023.09.1 software. We utilized the *meta* and *metafor* libraries and the generic inverse variance method (GIVM) with *Restricted Maximum-Likelihood* (REML) for tau^2^. As some studies do not report the number of cases and events, we performed the meta-analysis using the GIVM, requiring only the input of the effect measure (OR, RR, HR) and the 95% CI (Higgins et al., 2023). We applied the Hartung and Knapp correction, accounting for the uncertainty in estimating the variance between studies, which is more significant with a small number of studies (Bender et al., 2018; Knapp and Hartung, 2003; Harrer et al.). We considered RRs equivalent to the ORs if the frequency of the event of interest was <10% (McKenzie and Thomas, 2020). For studies reporting ORs or RRs stratified into different subgroups, we considered each subgroup analysis as a separate study.

Our protocol stated that we would examine heterogeneity among studies with Cochran’s Q test and Higgins I^2^ statistic, using a fixed effects model if heterogeneity were not statistically significant (*p* > 0.10, I^2^ statistics < 40%). On the other hand, we would use a random effects model (Higgins et al., 2023). The potential subgroups for analysis included study type, the continent of origin, drug type or class, dosage, and duration of exposure to CCB. We performed sensitivity and influence analyses, as well as metaregression, to assess heterogeneity.

### 2.5 Quality assessment

We assessed the risk of bias using the Newcastle–Ottawa scale (NOS) (Wells et al., 2023) and version 2 of the Cochrane risk-of-bias tool for randomized trials (ROB 2) (Higgins et al., 2011). We explored publication bias using funnel plots, Egger’s test, trim-and-fill analysis, and classic fail-safe N analysis.

### 2.6 GRADE assessment

Two researchers (MA-R and VB-Z) independently assessed the certainty of the evidence (CoE) of the study outcomes for each study outcome based on the Grading of Recommendations Assessment, Development, and Evaluation (GRADE) criteria (Granholm et al., 2019; GRADE, 2023). Any reviewer discrepancies were resolved through discussion with the lead researcher (EM-R).

## 3 Results

We identified 50 records, all retrieved from databases. After removing 18 documents, 32 reports remained. Subsequently, 14 reports were assessed for eligibility. Of these studies, 6 were excluded—mainly because they were narrative reviews or did not meet our PICO/PECO question (Supplementary Table S2). Finally, eight papers were included in our systematic review (Figure 1 and Table 1).

**TABLE 1:** General characteristics of included studies.

Study, year, country	Study design	Patients and events (cases)	Exposition	Outcome	Adjustment factors	aOR/aRR/aHR (95% CI)
Lee CC (Lee et al., 2021), 2021, Taiwan	NCC	Adults (≥18 years). Both sexes.NHIRD. Total 824,564, cases (TB) 8,164, and controls 816,400. Enrollment between January 1999 and December 2011. Follow-up 6.37 years	DHP-CCB (nifedipine, isradipine, nicardipine, felodipine, and amlodipine), No-DHP-CCB (verapamil, diltiazem) during the year prior to the TB diagnosis	Active TB.	Year of TB diagnosis, age, sex, risk score for TB disease	aRR 0.68 (0.58–0.78) for any CCB and active TB. aRR 0.63 (0.53–0.79) for DHP-CCB, compared to no use of CCBs and active TB. aRR 0.73 (0.57–0.94) for Non-DHP-CCB, compared to no use of CCBs, and active TB.
Lee C (Lee et al., 2015b), 2015, Taiwan	NCC	Adults. Both sexes. NHIRD. Total, one million patients, 7,164 new cases of active TB, and 716,400 controls. Enrollment between January 1997 and December 2011. Follow-up 13 years	DHP-CCB, phenylalkylamine, and benzodiazepine CCB. CCB exposure: received ≥ 7 days of prescription. Current use: prescription that ended within 30 days of the index date (first day of TB diagnosis)	Active TB	Risk score for disease (TB)	aRR 0.70 (0.64–0.77) for DHP-CCB and active TB. The dose-response analysis suggested that long-term use of DHP-CCB may further reduce the risk of active TB
Lee MY (Lee et al., 2015a), 2015, Taiwan	PCS	Adults (>65 years). Both sexes. NHIRD. Total one million patients, 50,645 with DM and 50,645 without DM. CCB users 17,240, CCB and DM 10,078. TB among those with DM 352, TB among those without DM 271. Enrollment from 1998 to 2009. Follow-up 12 years	They do not specify the type of CCB.	Active TB.	Age, sex, income, residence, gout, HT, hyperlipidemia, asthma, COPD, AIDS, connective tissue disease, ESKRD, HF, other cardiovascular diseases, antidiabetic medications, antihypertensives, lipid-lowering medications	aRR 0.76 (0.58–0.98) for CCB and active TB in diabetic patients >65 years old
Chidabaram V (Chidambaram et al., 2021), 2021, Taiwan	RCS	Adults (>18 years) treated for drug susceptible TB at the National Taiwan University Hospital (NTUH). Both sexes. Total of 2,894 cases of sensitive pulmonary TB. 36.4% had HT. Median age 66.6 years (IQR 49.1–77.8). Enrollment from 2000 to 2016. Follow-up 17 years	Minimum 2 weeks (14 doses) of CCB. DHP-CCB (amlodipine, felodipine, lercanidipine, nifedipine, nicardipine), Non-DHP-CCB (verapamil, diltiazem)	All-cause mortality and infection-related mortality during the first 9 months of TB treatment (composite outcome of death due to pneumonia, sepsis, or TB)	Sex, BMI, sputum AFB staining at diagnosis, cavitary disease, transplant history, CCI.	aHR 0.62 (0.34–1.13) for all CCBs and all-cause mortality. aHR 0.67 (0.37–1.21) for DHP-CCBs and all-cause mortality. aHR 0.70 (0.29–1.69) for all CCBs and infection-related mortality. aHR 0.76 (0.31–1.85) for DHP-CCBs and infection-related mortality
Chen HH, (Chen et al., 2020), 2020, Taiwan	RCS	Patients with diabetes >20 years old. NHIRD. DPP4i users: 6,399. Non-DPP4i users: 6,399. The incidence of TB in DPP4i users was 22.2 per 1,000 person-years, while in non-users, was 16.2 per 1,000 person-years. TB cases in non-users of DPP4i and users of CCB was 6. TB in users of DPP4i and CCB was 26. Enrollment between 2000 and 2012, end of the study 31 December 2013. Follow-up 5 years	DPP4 inhibitors and CCB (does not specify which one or the type)	Risk of developing TB.	Sex, age, DCSI score, all comorbidities, all medicines, including antihypertensives, insulinetc.	aHR 1.21 (0.60–2.44; *p* = 0.59) for TB among DPP4i users compared to non-DPP4i users and use of CCB compared to non-use of CCB.
Lee MC (Lee et al., 2018), 2018, Taiwan	RCS	Newly diagnosed patients with diabetes. NHIRD. There were 88,866 metformin users (>90 cumulative DDD in 1 year), and 88,866 non-users of MET matched by propensity score. TB 707 in MET users and 807 in MET non-users. Enrollment from January 2011 to December 2012. Follow-up: 18 years	Metformin, insulin, other oral antidiabetic medications, aspirin, CCBs (does not specify which one or the type), immunosuppressants, etc.	Incident TB (newly diagnosed tuberculosis) according to “validated” diagnostic criteria	Comorbidities: COPD, lung cancer, extrapulmonary cancer, cirrhosis, acquired immunodeficiencies, rheumatoid arthritisetc.	aHR 0.65 (0.51–0.82) for TB risk in metformin and CCB users, compared to non-users
Nawabooniyom K (Nawaboonniyom et al., 2021), 2021, Thailand	RCS	Both sexes. A total of 2,842 patients with pulmonary tuberculosis. CCB exposed 157 (5.5%), non-CCB exposed 2,685 (94.5%). Among the exposed 57 had diabetes, and among the non-CCB exposed 529 had diabetes mellitus. Enrollment from January 2013 through August 2017. Follow-up: not reported	CCB exposure: concurrent use of CCB during admission after starting anti-TB drugs, regardless of pre-admission CCB use. CCB (does not specify which one or the type)	90-day mortality in hospitalized patients with pulmonary TB.	Age, sex, comorbidities, intubation on admission, and medications, such as statins, beta-blockers, corticosteroids, aspirin, initial anti-TB regimen, etc.	The 90-day all-cause mortality were similar between exposed and non-CCB exposed (56 [35.7%] vs. 969 [36.1%]; aRR 0.999; 95% CI 0.981–1.018)
Lee MTG (Lee et al., 2014), 2014, Taiwan	NCC	Both sexes. NHIRD. One million patients, 7,164 new cases of active TB, and 716,400 controls. Follow-up: 13 years	CCB (does not specify which one or the type). CCB exposure: received ≥ 7 days of prescription. Current use: prescription that ended within 30 days of the index date (first day of TB diagnosis)	Active TB	Risk score for disease (TB)	aRR 0.72 (0.66–0.78) for TB risk in current CCB users. aRR 0.88 (0.77–0.99) for TB risk in recent CCB users

NCC, nested case-control study; PCS, prospective cohort study; RCS, retrospective cohort study; HT, hypertension; CCB, calcium channel blockers; DHP-CCB, dihydropyridine calcium channel blockers; Non-DHP-CCB, non-dihydropyridine calcium channel blockers; NHIRD, national health insurance research database; TB, tuberculosis; aOR/aRR/aHR, adjusted OR, RR, HR; CCI, Charlson comorbidity index; DCSI, diabetes complications severity index; DM, diabetes mellitus; ESCKD, End-Stage chronic kidney disease; HF, heart failure; COPD, chronic obstructive pulmonary disease; BMI, body mass index; AFB, acid-fast bacilli; DDD, defined daily dose; MET, metformin; LTBI, latent tuberculosis infection.

Among the eight studies included in this systematic review and meta-analysis, three were nested case-control, four were retrospective cohort, and one was a prospective cohort study. This review encompasses 4,020,830 patients, including 241,761 with diabetes mellitus and 30,397 with active tuberculosis (Table 1).

We only included articles that reported adjusted association measures (aOR, aRR, or aHR) and a control group. Four of the analyzed articles included patients with diabetes mellitus (Lee et al., 2015a; Lee et al., 2018; Chen et al., 2020; Nawaboonniyom et al., 2021), with one of them also investigating both patients with and without diabetes mellitus (Lee et al., 2015a; Nawaboonniyom et al., 2021), while two exclusively focused on patients with diabetes mellitus (Lee et al., 2018; Chen et al., 2020). All the included studies were conducted in Asia, and the follow-up period varied, with an average of 9.1 years (ranging from a minimum of 3 months to a maximum of 18 years).

The CCBs analyzed belonged to the dihydropyridine type (DHP-CCBs), including nifedipine, isradipine, nicardipine, felodipine, and amlodipine; and non-dihydropyridine (No-DHP-CCBs), which encompassed verapamil and diltiazem. Most studies did not report the CCB doses used. Five studies did not specify the type of CCB administered (Lee et al., 2014; Lee et al., 2015a; Lee et al., 2018; Chen et al., 2020; Nawaboonniyom et al., 2021). Since the study by Lee et al. (2021) reported RRs stratified into different subgroups (DHP-CCB and Non-DHP-CCB), we considered each subgroup analysis as a separate study for meta-analysis.

### 3.1 Risk of developing active tuberculosis and mortality

**FIGURE 2:**
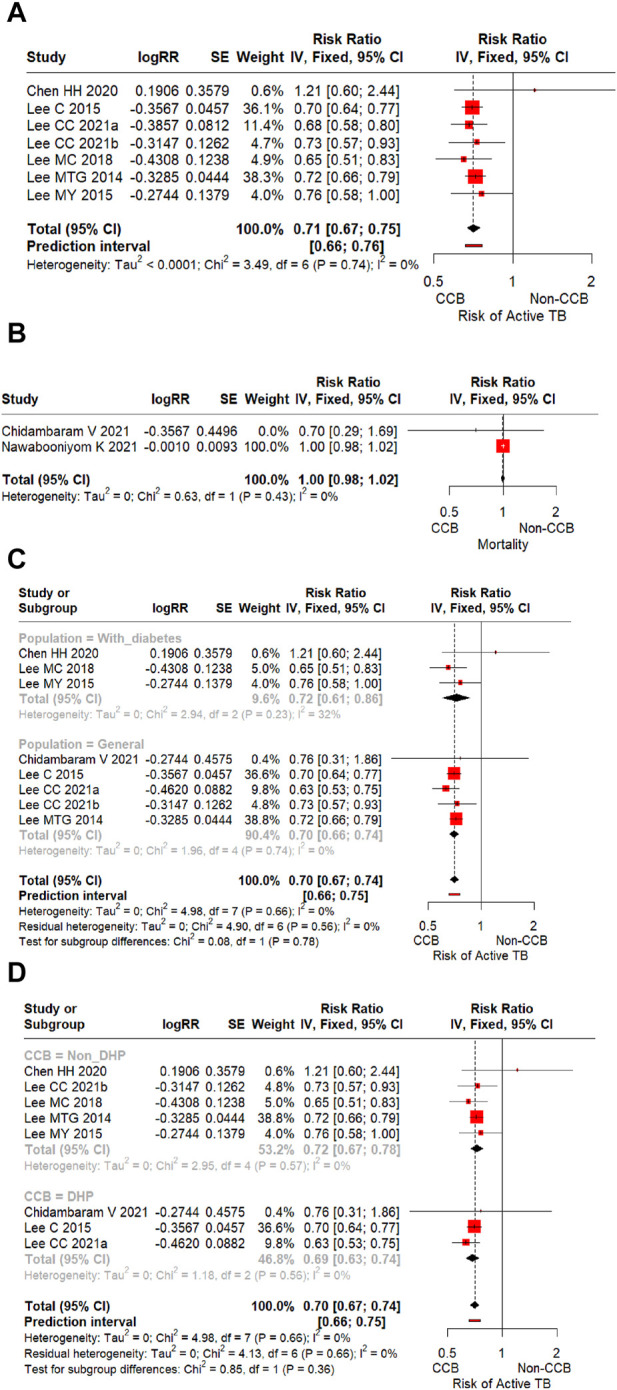
**(A)** *Forest plot* of the effect of CCBs on the risk of developing active tuberculosis in all patients (general population and patients with diabetes mellitus). **(B)** *Forest plot* of the effect of CCBs on tuberculosis-related mortality risk in all patients (general population and patients with diabetes mellitus). **(C)** *Forest plot* of the effect of CCBs on the risk of developing active tuberculosis according to the type of population (general population vs. patients with diabetes mellitus). **(D)** *Forest plot* of the effect of CCBs on the risk of developing active tuberculosis according to the class of CCB (dihydropyridines vs. non-dihydropyridines).

Based on our findings, CCBs reduce the risk of developing active tuberculosis by 29% (RR 0.71; 95% CI 0.67–0.75) in patients with and without diabetes mellitus (Figure 2A). Statistical heterogeneity was not significant for this outcome (I^2^ = 0, *p* = 0.74). On the contrary, CCBs did not show any benefit in the risk of mortality related to tuberculosis (RR 1.00; 95% CI 0.98–1.02) (Figure 2B). For this outcome, no statistical heterogeneity was found (I^2^ = 0, *p* = 0.43). However, it is noteworthy that we found only two studies exploring tuberculosis-related mortality (Chidambaram et al., 2021; Nawaboonniyom et al., 2021).

### 3.2 Heterogeneity

Statistical heterogeneity was not significant (I^2^ = 0%, *p* > 0.1) for either outcome. The sensitivity analysis and the leave-one-out test did not significantly impact the overall estimate (Supplementary Figure S1). Furthermore, we performed meta-regression analyses to explore potential sources of between-study heterogeneity. The moderators’ test was not statistically significant according to the type of population (patients with diabetes vs. general population) (QM = 0.0813, *p* = 0.78) or the CCB class (DHP-CCBs vs. non-NHP-CCBs) (QM = 0.548, *p* = 0.36). In other words, none of these variables accounted for variability between studies. Similarly, the test for residual heterogeneity was also not statistically significant for any of these variables (QE = 4.8997, *p* = 0.57 and QE = 4.1262, *p* = 0.67, respectively), suggesting that our model was well specified. Then, no other moderating variables would be considered in our model (Supplementary Table S3).

Subgroup analysis showed that the protective effect of CCBs on the risk of developing active tuberculosis was present in patients with diabetes mellitus and the general population (test for subgroup differences I^2^ = 0, *p* = 0.78). This beneficial effect was more significant among the general population (RR 0.70; 95% CI 0.66–0.74) compared to patients with diabetes mellitus (RR 0.72; 95% CI 0.61–0.86) (Figure 2C). Similarly, the protective effect of CCBs on the risk of active tuberculosis was irrespective of the class of the CCB (test for subgroup differences I^2^ = 0, *p* = 0.36). However, this beneficial effect was more important among those patients treated with DHP-CCBs (RR 0.69; 95% CI 0.63–0.74) compared to patients treated with non-DHP-CCBs (RR 0.72; 95% CI 0.67–0.78) (Figure 2D).

### 3.3 Publication bias

**FIGURE 3:**
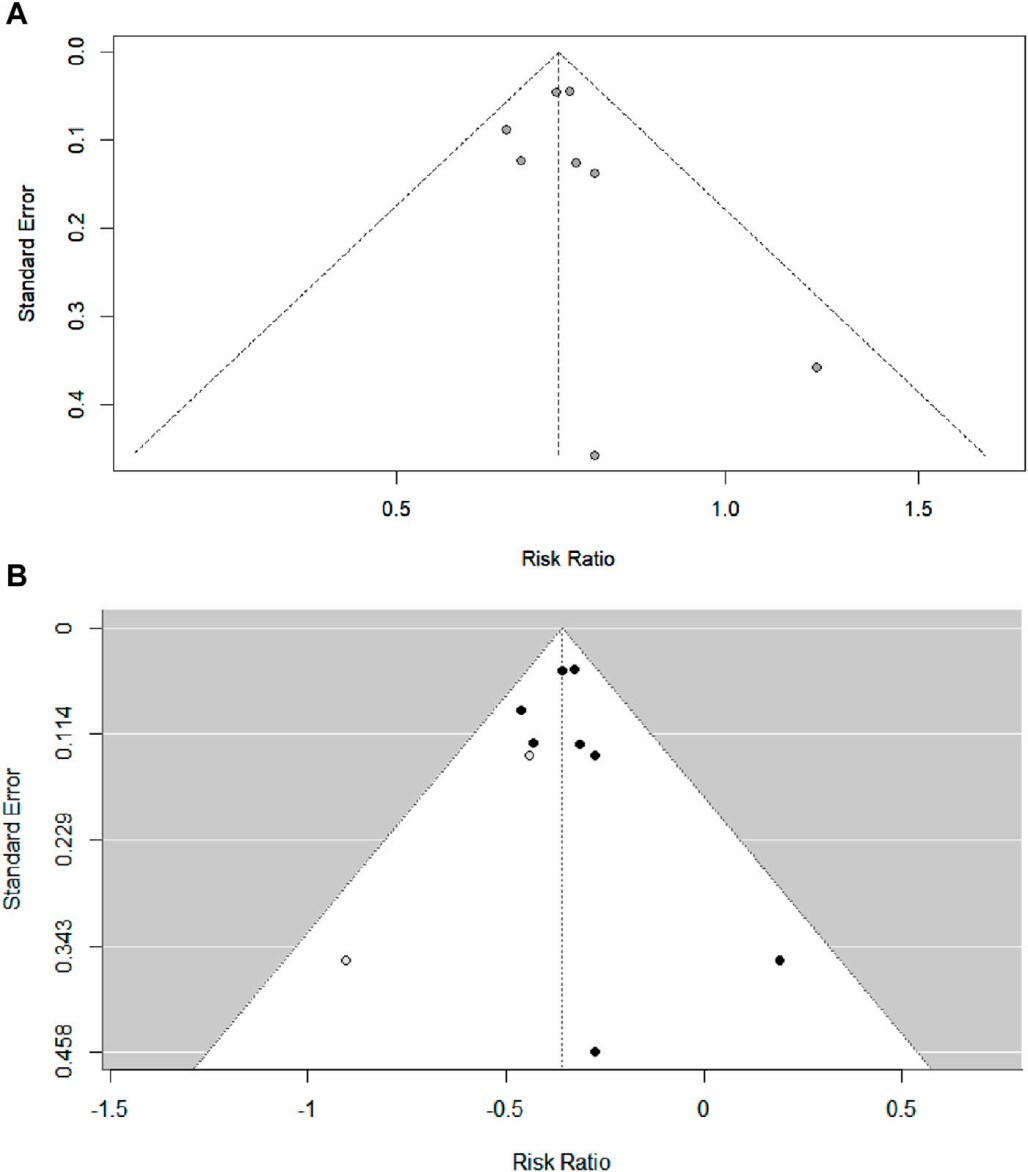
**(A)** Classic *funnel plot* of the included studies of the effect of CCBs on the risk of developing active tuberculosis. **(B)** Trim-and-fill analysis of the included studies of the effect of CCBs on the risk of developing active tuberculosis.

Even though our funnel plot did not suggest a risk of publication bias (Figure 3A), we conducted an Egger’s test, trim-and-fill, and a classic fail-safe N analysis. Egger’s test did not show a risk of publication bias (*z* = 0.62, *p* = 0.5375). Likewise, considering a reference or threat criterion (5 * *k* + 10 = 50), the Rosenthal approach (observed significance level *p* < 0.0001, target significance level *p* = 0.05, Fail-safe *N* = 292) and the Rosenberg approach (observed significance level *p* < 0.0001, target significance level *p* = 0.1, Fail-safe *N* = 469) suggested that publication bias was not a threat to the existence of a significant effect size in this meta-analysis. The trim-and-fill plot concurred with the other publication bias analyses commented on above (Figure 3B).

Performing a duration and dose-response analysis was not possible because most studies, except two (Lee et al., 2021; Lee et al., 2015b), did not report the doses or duration of exposure to CCBs. Nevertheless, one of these studies suggested that longer-term dihydropyridines can lead to an even lower risk of active tuberculosis (Lee et al., 2015b). However, the authors did not specify the exact time and dose of CCB. The other study was the only one that reported the dose and duration of use of CCB (Lee et al., 2021). Five studies did not report the type of CCB administered (Lee et al., 2014; Lee et al., 2015a; Lee et al., 2018; Chen et al., 2020; Nawaboonniyom et al., 2021).

All included studies had a low risk of bias (Table 2).

**TABLE 2:** Risk of bias of the included studies according to NOS tool.

Author, study, country	Study design	Selection	Comparability	Outcome	Total	Conclusion
Lee CC (Lee et al., 2021), 2021, Taiwan	NCC	***	**	***	8	Low risk
Lee C (Lee et al., 2015b), 2015, Taiwan	NCC	**	**	***	7	Low risk
Lee MY (Lee et al., 2015a), 2015, Taiwan	PCS	***	**	***	8	Low risk
Chidambaram V (Chidambaram et al., 2021), 2021, Taiwan	RCS	***	***	***	9	Low risk
Chen HH, (Chen et al., 2020), 2020, Taiwan	RCS	***	**	***	8	Low risk
Lee MC (Lee et al., 2018), 2018, Taiwan	RCS	***	***	***	9	Low risk
Lee MTG (Lee et al., 2014), 2014, Taiwan	NCC	**	**	***	7	Low risk
Nawaboonniyom K (Nawaboonniyom et al., 2021), 2021, Thailand	RCS	***	**	***	8	Low risk

NCC, nested case control study; PCS, prospective cohort study; RCS, retrospective cohort study.

Note: An asterisk (*) represents a star in each domain of the Newcastle–Ottawa scale (NOS) tool.

### 3.4 GRADE assessment

We upgraded the level of CoE as all the studies included exhib-ited a low risk of bias. Indirectness (the included studies compared similar interventions, similar populations, and similar outcomes), imprecision (this review encompasses 4,020,830 patients, 241,761 individuals with diabetes mellitus, and 30,397 patients with active tuberculosis), publication bias, and inconsistency (I^2^ = 0) did not impact signifi-cantly the CoE. Consequently, we judged the CoE using the GRADE criteria as moderate.

## 4 Discussion

### 4.1 Risk of developing active tuberculosis and mortality

Based on our findings, CCBs reduce the risk of developing active tuberculosis by 29% (RR 0.71; 95% CI 0.67–0.75) in patients with and without diabetes mellitus (Figure 2A). On the contrary, CCBs did not show any benefit in the risk of mortality related to tuberculosis (RR 1.00; 95% CI 0.98–1.02) (Figure 2B). For this outcome, no statistical heterogeneity was found (I^2^ = 0, *p* = 0.43). However, it is noteworthy that we found only two studies exploring tuberculosis-related mortality (Chidambaram et al., 2021; Nawaboonniyom et al., 2021).

Although statistical heterogeneity was not significant (I^2^ = 0%, *p* > 0.10) in both outcomes evaluated, we conducted subgroup analyses to explore the effect of CCBs on the risk of active tuberculosis according to the type of population (patient with diabetes vs. general population) (Figure 2C) and the pharmacological class of CCB administered (DHP-CCB vs. non-DHP-CCB) (Figure 2D). This analysis showed that the protective effect of CCBs is lower in the subgroup of patients with diabetes (RR 0.72; 95% CI 0.61–0.86) compared to the general population (RR 0.70; 95% CI 0.66–0.74), and it is also lower in the subgroup of patients who used non-DHP-CCBs (RR 0.72; 95% CI 0.67–0.78), compared to those who used DHP-CCBs (RR 0.69; 95% CI 0.63–0.74). However, these differences were not statistically significant (I^2^ = 0, *p* > 0.10). In addition, the sensitivity analysis, excluding outliers, did not significantly affect the overall estimate, suggesting good consistency between individual studies (Bender et al., 2018; Lee et al., 2018). Similarly, meta-regression analyses indicated that our model was well-specified, and no other moderating variables were overlooked in our analysis.

This study is the first systematic review and meta-analysis to assess the effect of CCBs on the risk of incident tuberculosis and mortality from tuberculosis. Therefore, it is not possible to compare our findings with other similar studies. However, our results are consistent with most published primary studies that evaluated the outcome of incident active tuberculosis risk (Lee et al., 2021; Lee et al., 2014; Lee et al., 2015a; Lee et al., 2015b; Lee et al., 2018). Only one study found no benefit of CCB on the risk of developing active tuberculosis (Chen et al., 2020).

Typically, meta-regression and subgroup analysis are unnecessary when heterogeneity is zero. However, in some cases, even with zero heterogeneity, a meta-regression analysis may be beneficial (Higgins and Thompson, 2002; Higgins et al., 2003; Borenstein et al., 2009; Jpt). For example, treatment-effect relationships with other variables. Another reason for conducting a meta-regression is that I^2^ has a substantial bias when the number of studies is small (von Hippel, 2015). The bias is positive when the true fraction of heterogeneity is small, but the bias is typically negative when the true fraction of heterogeneity is large. For example, with seven studies and no true heterogeneity, I^2^ will overestimate heterogeneity by an average of 12%. Even further, with seven studies and 80% true heterogeneity, I^2^ can underestimate heterogeneity by an average of 28%. Biases of 12%–28% are not trivial since, in the Cochrane Library, the median I^2^ estimate is 21% (von Hippel, 2015; Ioannidis et al., 2007).

None of these primary studies that examined tuberculosis related mortality showed a benefit of CCBs on mortality (Chidambaram et al., 2021; Nawaboonniyom et al., 2021). Chidambaram et al. (2021) included adult patients with culture-confirmed susceptible tuberculosis. The authors reported that in the multivariate analysis, after adjustment for confounders (age and prior tuberculosis), all-CCB did not reduce 9-month all-cause mortality (aHR 0.62; 95% CI 0.34–1.13) or 9-month infection-related mortality (aHR 0.70; 95% CI 0.29–1.69). Similarly, after adjustment for the same confounders, DHP-CCB did not reduce 9-month all-cause mortality (aHR 0.67; 95% CI 0.37–1.21) or 9-month infection-related mortality (aHR 0.76; 95% CI 0.31–1.85). Notably, out of the 1,052 hypertensive patients included, 78% were older than 65, with the median age being 75.53 years (IQR 66.55–82.43). Furthermore, 72.2% were men, and 41.2% of these hypertensive patients smoked. All of these conditions are independent factors associated with increased risk of mortality. Therefore, the authors concluded that DHP-CCBs may lower all-cause mortality in TB patients with hypertension.

In the same way, the study conducted by Nawaboonniyom et al. (2021) failed to demonstrate a positive impact of CCBs on tuberculosis-related mortality. However, this study differed from all the others because it is the only one that examined the association between CCBs and mortality in hospitalized patients with pulmonary tuberculosis. As expected, these inpatients had a high risk of mortality. Indeed, the authors stated that older age, intubation on admission, initiation of a 3-drug anti-tuberculosis regimen (compared to a 4-drug anti-tuberculosis regimen), and proton pump inhibitors were associated with higher 90-day all-cause mortality. Furthermore, the group of CCB users, when compared to non-CCB users, had more hypertensive and more patients with diabetes, although these differences did not reach statistical significance (aHR 1.269; 95% CI 0.979–1.644 and aHR 1.066; 95% CI 0.852–1.334, respectively).

Currently, anti-tuberculosis therapy alone cannot overcome the challenges of tuberculosis treatment (Lee et al., 2015a). Tuberculosis treatment involves the prolonged and simultaneous administration of drugs that could cause significant adverse effects and discontinuation (Munro et al., 2007; Bea et al., 2021). Additionally, in up to 25% of cases, MTB is resistant to chemotherapy. Consequently, discovering new methods of prevention and treatment for this disease is a top priority for public health and research (Barba Evia, 2020).

There is recent evidence that CCBs, beta-blockers, statins, and oral antidiabetics could act as immunomodulators—by improving the host’s anti-mycobacterial responses—and optimize the effect of anti-tuberculosis drugs or reducing the risk of developing active tuberculosis (Kristiansen and Amaral, 1997; Gupta et al., 2009; Gupta et al., 2013; Meregildo-Rodriguez et al., 2022a; Meregildo-Rodriguez et al., 2022b), which is consistent with our results. Conversely, one systematic review and meta-analysis has reported that some oral antidiabetic drugs, such as DPP-4 inhibitors, could increase the risk of developing tuberculosis in patients with diabetes (Meregildo-Rodriguez et al., 2022b).

We highlight the following strengths of this study: 1) it is the first systematic review and meta-analysis assessing the effect of CCBs on the risk of active tuberculosis and tuberculosis-related mortality; 2) our search strategy was broad, comprehensive and included all published studies to date; 3) we only included studies that reported adjusted effect sizes; 4) we only included primary studies that specifically examined clinical outcomes, not intermediate outcomes; 5) we conducted sensitivity, subgroup and meta-regression analyses; and 6) we found no significant heterogeneity, publication bias, or risk of bias. Therefore, our results are robust and consistent with the available primary studies. One issue with pooling raw effect sizes in a meta-analysis of non-randomized observational studies is that it provides no more information than a univariate analysis of the original observational studies (Liu et al., 2017; Paul and Leeflang, 2021). The Cochrane Handbook advises adopting the adjusted model estimate with the greatest number of confounding variables (Higgins et al., 2023), as combining uncorrected data may result in the observation of a significant impact that may be diminished or even eliminated when controlled for these covariates (Higgins et al., 2003).

However, this study also has some crucial limitations, primarily stemming from the small number of studies: 1) all included studies were conducted on a single continent, which limits the generalizability of our findings to non-Asian populations; 2) since at least 6 of 7 studies conducted in Taiwan were performed by using the Taiwan National Health Insurance Research Databases (Lee et al., 2021; Lee et al., 2015a; Lee et al., 2015b) it is possible that there was a lot of overlapping for patients’ selection; 3) it was not possible to conduct a duration and dose-response analysis because most studies did not report the doses, the time, or the specific type of CCB prescribed. Nonetheless, one study suggested that only patients using CCBs for more than 90 days had a lower risk of active tuberculosis (Lee et al., 2021).

## 5 Conclusion

Calcium channel blockers may significantly reduce the risk of developing active tuberculosis by up to 29%, both in patients with diabetes and in the general population. This protective effect seems to be more significant in the general population compared to patients with diabetes and with the use of DHP-CCB compared to non-DHP-CCBs. Conversely, CCBs do not appear to reduce tuberculosis-related mortality. However, further studies are needed to validate our results before recommending CCBs to cardiovascular patients at high risk of TB. In addition, some aspects remain to be clarified, such as the dose and the time from which CCBs exert this “protective” effect on the risk of suffering from active tuberculosis.

## Data Availability

The raw data supporting the conclusion of this article will be made available by the authors, without undue reservation.
