# Supplementary material for: Effect of calcium-channel blockers on the risk of active tuberculosis and mortality: systematic review and meta-analysis
Source: Front Pharmacol. 2024 Jan 18;15:1298919. doi: 10.3389/fphar.2024.1298919 (PMC10830796; doi:10.3389/fphar.2024.1298919)

| **Table S1.** Search strategy | | | |  |  |
| --- | --- | --- | --- | --- | --- |
| **1. MEDLINE VIA PUBMED** | | | | Results | Date |
| #1 | | | "Tuberculosis"[Mesh] | 202,653 | 30/September/2023 |
| #2 | | | "Calcium Channel Blockers"[Mesh] | 37,470 |  |
| #3 | | | #1 AND #2 | 16 |  |
| **2. GOOGLE SCHOLAR** | | | | Results | Date |
| #1 | | | All in title "active tuberculosis" | 9,880 | 30/September/2023 |
| #2 | | | All in title "calcium channel blocker" | 15,400 |  |
| #3 | | | #1 AND #2 | 56 |  |
| **3. SCOPUS** | | | | Results | Date |
| #1 | | | TITLE-ABS-KEY ('tuberculosis') | 345,893 | 30/September/2023 |
| #2 | | | TITLE-ABS-KEY ('calcium channel blocker') | 62,009 |  |
| #3 | | | #1 AND #2 | 43 |  |
| **4. EMBASE** | | | | Results | Date |
| #1 | 'tuberculosis'/exp | | | 318,029 | 30/September/2023 |
| #2 | 'Calcium channel blocking agent'/exp | | | 68,575 |  |
| #3 | #1 AND #2 | | | 936 |  |
| #4 | #1 AND #2 AND #3 AND ('article'/it OR 'article in press'/it OR 'conference abstract'/it OR 'conference paper'/it OR 'editorial'/it OR 'letter'/it OR 'review'/it) AND ('clinical article'/de OR 'clinical trial'/de OR 'cohort analysis'/de OR 'controlled study'/de OR 'cross sectional study'/de OR 'human'/de OR 'major clinical study'/de OR 'observational study'/de OR 'prospective study'/de OR 'retrospective study'/de OR 'systematic review'/de) | | | 49 |  |
| **5. WEB OF SCIENCE** | | | | Results | Date |
| #1 | | "Tuberculosis" | | 84,500 | 30/September/2023 |
| #2 | | "Calcium channel blocker” | | 1,528 |  |
| #3 | | #1 AND #2 | | 1 |  |
| **6. SCIENCEDIRECT** | | | | Results | Date |
| #1 | | "Tuberculosis" | | 10,211 | 30/September/2023 |
| #2 | | "Calcium channel blocker" | | 67,026 |  |
| #3 | | #1 AND #2 | | 35 |  |

| **Table S2.** Excluded Studies and the reason for their exclusion |  |
| --- | --- |
| **Study** | **Reason for exclusion** |
| Song L, Cui R, Yang Y, Wu X. Role of calcium channels in cellular antituberculosis effects: Potential of voltage-gated calcium-channel blockers in tuberculosis therapy. J Microbiol Immunol Infect. 2015 Oct;48(5):471-6. doi: 10.1016/j.jmii.2014.08.026. | Review. |
| Mitini-Nkhoma SC, Chimbayo ET, Mzinza DT, Mhango DV, Chirambo AP, Mandalasi C, Lakudzala AE, Tembo DL, Jambo KC, Mwandumba HC. Something Old, Something New: Ion Channel Blockers as Potential Anti-Tuberculosis Agents. Front Immunol. 2021 Jun 24;12:665785. doi: 10.3389/fimmu.2021.665785. | Review. |
| D'Elia JA, Weinrauch LA. Gated Calcium Ion Channel and Mutation Mechanisms in Multidrug-Resistant Tuberculosis. Int J Mol Sci. 2023 Jun 2;24(11):9670. doi: 10.3390/ijms24119670. | Review. |
| Wu JY, Lee MG, Lee SH, Lee SH, Tsai YW, Hsu SC, Chang SS, Lee CC. Angiotensin-Converting Enzyme Inhibitors and Active Tuberculosis: A Population-Based Study. Medicine (Baltimore). 2016 May;95(19):e3579. doi: 10.1097/MD.0000000000003579. | Outcome different from our PECO / PICO question. |
| Kazibwe A, Kyazze AP, Ssekamatte P, Mutebi EI, Kibirige D, Kaddumukasa M, Kiguli S, Andia-Biraro I. TB infection in adults with diabetes mellitus in Uganda. Int J Tuberc Lung Dis. 2023 Apr 1;27(4):308-314. doi: 10.5588/ijtld.22.0614. | Outcome different from our PECO / PICO question. |
| D'Elia JA, Weinrauch LA. Calcium Ion Channels: Roles in Infection and Sepsis Mechanisms of Calcium Channel Blocker Benefits in Immunocompromised Patients at Risk for Infection. Int J Mol Sci. 2018 Aug 21;19(9):2465. doi: 10.3390/ijms19092465. PMID: 30134544; PMCID: PMC6164603. | Population different from our PECO / PICO question. |

| **Table S3.** Characteristics of study participants and cases of the included studies | | | | | |
| --- | --- | --- | --- | --- | --- |
| Study, year, country | Study design | Participants and events (cases) | Ethnic group | Comorbidities and demographic characteristics | Other important outcomes |
| Lee CC [17], 2021, Taiwan | NCC | Adults (≥18 years). Both sexes. NHIRD. Total 824,564, cases (TB) 8,164, and controls 816,400. Non-CCB (n = 36,643), DHP-CCB (n= 69,200), Nonusers (n = 717, 473). Male: 26,054 (71.10%), 47, 779 (69.04%), 491, 584 (68.52%), respectively. Enrollment between January 1999 and December 2011. Follow-up 6.37 years. | Asian (Taiwanese population) | Baseline combined comorbidity score 2.38±2.41, 1.80±2.24, and 0.96±1.64, respectively for Non-CCB, DHP-CCB, and nonusers.  Diabetes without chronic complications 12,151 (33.16%), 24,578 (35.52), and 108 412 (15.11) (p < 0.001) respectively for Non-CCB, DHP-CCB, and nonusers.  Diabetes with chronic complications 4349 (11.87%), 8,975 (12.97%), and 32,060 (4.47) (p < 0.001) respectively for Non-CCB, DHP-CCB, and nonusers. AIDS/HIV 26 (0.07%), 22 (0.03%), 366 (0.05%) (p = 0.01) respectively for Non-CCB, DHP-CCB, and nonusers.  Other comorbidities reported: peripheral vascular disease, HF, liver diasease, cerebrovascular disease, myocardial infarction, dementia, COPD, renal disease, cancer, etc. | The disease risk score–adjusted risk of tuberculosis (TB) in other patient subgroups:  Patients without diabetes: 0.60 (95% CI 0.51-0.70). Patients with diabetes: 0.72 (95% CI 0.60-0.87). Patients without obesity: 0.64 (0.57-0.72).  Patients with obesity: 1.24 (0.42-3.67). |
| Lee C [24], 2015, Taiwan | NCC | Adults. Both sexes. NHIRD. Total, one million patients, 7,164 new cases of active TB, and 716,400 controls.  Enrollment between January 1997 and December 2011. Follow-up 13 years. | Asian (Taiwanese population) | Not reported. | Use of DHP-CCBs, but not phenylalkylamine and benzothiazepine CCBs were associated with lower risk of active TB. Current use of DHP-CCB was associated with lower risk of active TB before (RR 0.75; 95% CI, 0.69 – 0.82) and after DRS adjustment (RR 0.70; 95% CI, 0.64 – 0.77). Dose response analysis suggested that longer term use of dihydropyridine can lead to even lower risk of active TB. |
| Lee MY [8], 2015, Taiwan | PCS | Adults. Both sexes. NHIRD. Total one million participants, 50,645 with DM and 50,645 without DM. CCB users 17,240, CCB & DM 10,078. TB among those with DM 352, TB among those without DM 271. Male: 12,875, 6438 (46.0%), 6437 (46.1%) (p = 0.9919) for total participants, with diabetes mellitus, and without diabetes mellitus, respectively. Enrollment from 1998 to 2009. Follow-up 12 years. | Asian (Taiwanese population) | AIDS: 1, 1 (0.01%), and 0 (0.00%) (p = 0.3174) for total participants, with diabetes mellitus, and without diabetes mellitus, respectively.  ESCKD: 32, 204 (1.5%), and 125 (0.9%) (p = <0.0001) for total participants, with diabetes mellitus, and without diabetes mellitus, respectively.  Other comorbidities reported: gout, HT, hyperlipidemia, asthma, COPD, HF, other cardiovascular disease, and connective tissue disease. | Tuberculosis: 623, 352 (2.6%), and 271 (2.0%) (p = 0.0006) for total participants, with diabetes mellitus, and without diabetes mellitus, respectively. |
| Chidabaram V [25], 2021, Taiwan | RCS | Adults (>18 years) treated for drug susceptible TB at the National Tai-wan University Hospital (NTUH). Both sexes. Total of 2,894 cases of sensitive pulmonary TB. 36.4% had HT. Median age 66.6 years (IQR 49.1–77.8). Enrollment from 2000 to 2016. Follow-up 17 years. | Asian (Taiwanese population) | There were a higher proportion of patients aged ≥65 years in the group (78.0%) with HT relative to the normotensive group (37.7%; p < .001). Approx. 40% of the total patient population were ever smokers, and 2.8% reported alcoholism, with no significant differences between patients with and without HT. There were higher rates of DM, cardiovascular diseases, cerebrovascular accidents, cancer, CKD, COPD, and cirrhosis among patients in the hypertensive group. | During the first 9 months after TB treatment initiation, 303/2667 patients (11.4%) died of infection-related causes, constituting 55.7% (303/544 patients) of all deaths during the first 9 months of TB treatment. Of this group, 43 patients (14.2%) died due to TB-related causes. HT had significantly earlier infection-related mortality (p < .001). After adjusting for confounders, patients with HT had a 1.87 times higher hazard of infection-related mortality during TB treatment (95% CI, 1.34–2.61; p < .001). |
| Chen HH, [37], 2020, Taiwan | RCS | Patients with diabetes >20 years old. HIRD. DPP4i users: 6,399. Non-DPP4i users: 6,399. Enrollment between 2000 and 2012, end of the study December 31, 2013. Follow-up 5 years. | Asian (Taiwanese population) | Participants were mainly 40–59 years old. The mean age of DPP4i users was 61.9±13.3 years, and that of non-users was 62.3±12.7 years. Most participants had a DCSI score of >4.  Male: 3,288 (51.4%), 3,261 (51.0%) for non-users and users (p = 0.63), respectively.  Chronic kidney disease: 1,732 (27.1%), 1,752 (27.4%) for non-users and users (p = 0.69), respectively.  Other comorbidities reported: CAD, stroke, HT, hyperlipidemia, and COPD.  HIV/AIDS was not reported. | The incidence of TB in DPP4i users was 22.2 per 1,000 person-years, while in non-users, was 16.2 per 1,000 person-years. TB cases in non-users of DPP4i and users of CCB was 6. TB in users of DPP4i and CCB was 26.  When regarding DPP4i non-users as a reference group, the risk of developing TB in patients with more than 20 average DDDs per year increased by 2.19 times (p = 0.048). Patients with less than 20 average DDDs per year exhibited no effect on TB. |
| Lee MC [38], 2018, Taiwan. | RCS | Newly diagnosed patients with diabetes. NHIRD. 88,866 metformin users (>90 cumulative DDD in 1 year), and 88,866 non-users of MET matched by propensity score. TB 707 in MET users and 807 in MET non-users. Follow-up: 18 years. | Asian (Taiwanese population) | Age (mean ± SD): 55.9±13.1 and 55.9±12.9 years for metformin nonusers and metformin users (p = 0.697), respectively.  Male: 48, 70 (54.3%) and 48,345 (54.4%) for metformin nonusers and metformin users (p = 0.724), respectively.  Type 1 DM 1,920 (2.2%) and 1905 (2.1%) for metformin nonusers and metformin users (p = 0.819), respectively.  HIV/AIDS: 48 (0.05%) and 40 (0.04%) for metformin nonusers and metformin users (p = 0.819), respectively.  Other comorbidities reported: COPD, pulmonary cancer, extrapulmonary cancer, DM chronic complication, etc. | The male sex (HR: 2.90 [2.39–3.52]), and type 1DM (HR: 3.68 [2.03–6.68]) were the most potent risk factors for developing TB. Other independent risk factors included age (HR: 1.046 [1.034–1.058] per year increment), COPD (HR: 1.82 [1.27–2.61]), insulin use (HR: 2.03 [1.43–2.89]), NSAIDs use (HR: 1.38 [1.03–1.86]) and steroid use (HR: 1.75 [1.47–2.07]). |
| Nawabooniyom K [39], 2021, Thailand. | RCS | Both sexes. A total of 2,842 patients with pulmonary tuberculosis. CCB exposed 157 (5.5%), non-CCB exposed 2,685 (94.5%). Among the exposed 57 had diabetes, and among the non-CCB exposed 529 had diabetes. Enrollment from January 2013 through August 2017. Follow-up: not reported. | Asian (Thai population) | According to exposure or not exposure to CCBs, the former was relatively older (p < 0.001) and had a higher body mass index (p < 0.001), a larger proportion of patients with coexisting conditions including diabetes mellitus (p < 0.001), hypertension (p < 0.001).  HIV/AIDS was not reported. | Multivariate analysis showed the significant association between higher mortality rate and old age (AHR, 1.025; 95% CI, 1.019-1.031), intubation on admission (AHR, 1.639; 95% CI, 1.291-2.080), and initial treatment with three anti-TB regimens (AHR, 1.432; 95% CI, 1.099-1.864). Initiation of quadruple regimen reduced mortality rate (AHR, 0.754; 95% CI, 0.611 to 0.924). |
| Lee MTG [40], 2014, Taiwan | NCC | Adults. Both sexes. NHIRD. One million patients, 7,164 new cases of active TB, and 716,400 controls. Enrollment between January 1997 and December 2011. Follow-up 13 years. | Asian (Taiwanese population) | Not reported. | Decrease in the risk of active TB is inversely proportional to the time from the last use of CCBs. Conversely, past use of CCBs had no statistically significant influence on risk of active TB. |
| NCC: nested case-control study, PCS: prospective cohort study, CRS: retrospective cohort study, CSS: cross-sectional study, HT: hypertension, CCB: calcium channel blockers, DHP-CCB: dihydropyridine calcium channel blockers, Non-DHP-CCB: non-dihydropyridine calcium channel blockers, NHI: National Health Insurance, TB: tuberculosis, CCI: Charlson comorbidity index, DCSI: diabetes complications severity index, DM: Diabetes Mellitus, CAD: coronary artery disease, ESCKD: End-Stage chronic kidney disease, HF: heart failure, COPD: Chronic Obstructive Pulmonary Disease, BMI: body mass index, AFB: acid-fast bacilli, DDD: Defined Daily Dose, MET: metformin, LTBI: latent tuberculosis infection. DRS: disease risk score. | | | | | |

| **Table S4.** Meta-regression analysis of the studies included in the metanalysis assessing the effect of CCBs on the risk of developing active tuberculosis in all participants (general population vs. participants with diabetes mellitus). | | |
| --- | --- | --- |
| **Variable** | **Test of moderators (coefficient 2)** | **Test for residual heterogeneity** |
| ***Population*** (participants with diabetes vs. the general population). | QM (df = 1) = 0.0813, p = 0.7755 | QE (df = 6) = 4.8997, p = 0.5567 |
| **CCB class** (dihydropyridines vs. non-dihydropyridines). | QM (df = 1) = 0.548, p = 0.3552 | QE (df = 6) = 4.1262, p = 0.6596 |

**Figure S1a.** Influence, sensitivity and leave-one-out analysis of the studies included in the metanalysis assessing the effect of CCBs on the risk of developing active tuberculosis in all participants (general population vs. participants with diabetes mellitus).


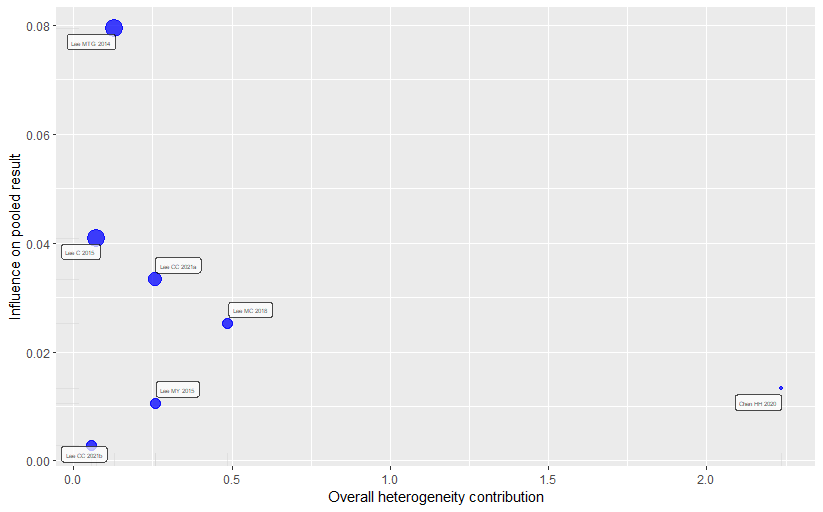


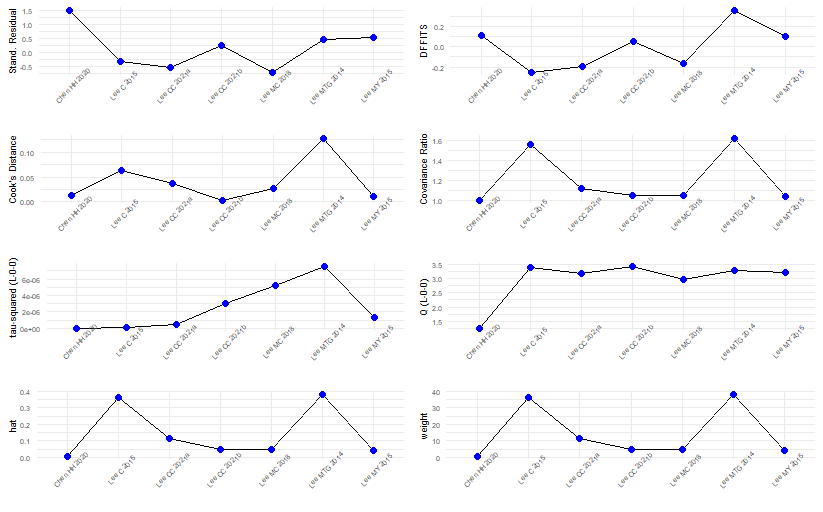


Leave-one-out analysis sorted by effect size


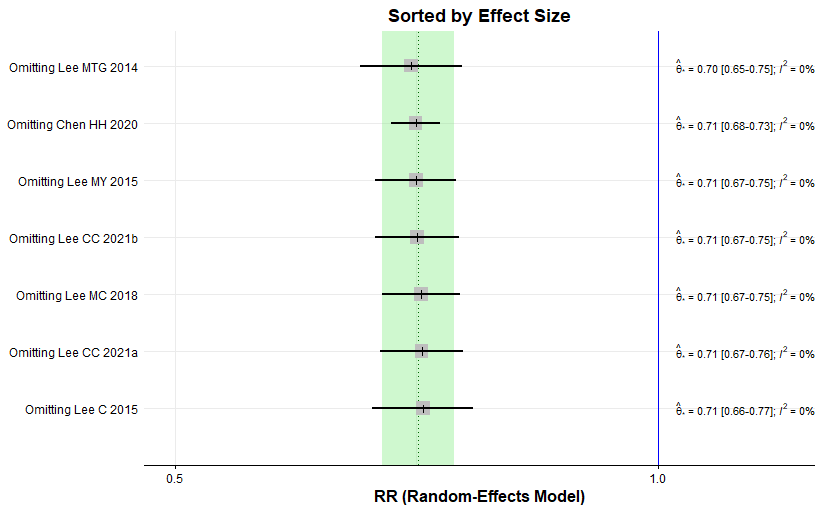


Leave-one-out analysis sorted by I^2^.


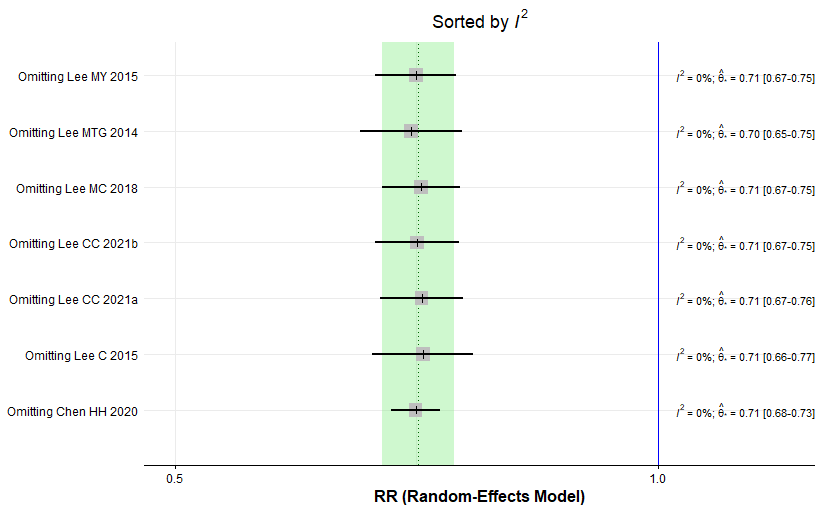

Supplement: Supplementary file 1 [file DataSheet2.docx]
